# Recognition of HER2 expression in hepatocellular carcinoma and its significance in postoperative tumor recurrence

**DOI:** 10.1002/cam4.2006

**Published:** 2019-02-04

**Authors:** Ji‐Hua Shi, Wen‐Zhi Guo, Yang Jin, Hua-Peng Zhang, Chun Pang, Jie Li, Pål‐Dag Line, Shui‐Jun Zhang

**Affiliations:** ^1^ Department of Hepatobiliary and Pancreatic Surgery, Henan Key Laboratory of Digestive Organ transplantation, The First Affiliated Hospital of Zhengzhou University Zhengzhou University Zhengzhou China; ^2^ Department of Transplantation Medicine, Institute of Surgical Research Oslo University Hospital Oslo Norway; ^3^ Department of Biosciences University of Oslo Oslo Norway; ^4^ Faculty of Medicine, Institute of Clinical Medicine University of Oslo Oslo Norway

**Keywords:** epithelial‐to‐mesenchymal transition, hepatocellular carcinoma, human epidermal growth factor receptor 2, Trastuzumab, tumor recurrence

## Abstract

**Background:**

The ERBB2 oncogene hypothesis is challenged in hepatocellular carcinoma (HCC) with the conflicting evidences of human epidermal growth factor receptor 2 (HER2) overexpression. HER2 could be a new target as a treatment option for HCC as well as tumor recurrence after surgery. HER2 in HCC biology needs further explorations.

**Methods:**

Clinical and mRNA data of HCC patients were obtained from TCGA HCC cohort, GSE89377 and GSE115018. Western Blotting and immunohistochemistry were employed to test expression of HER2, E‐cadherin, and Vimentin. In HepG2, JM1, HER2‐transfected McA cells, and TGF‐β cocultured JM1 cells, HCC biology, including cell survival, proliferation, and epithelial‐to‐mesenchymal transition (EMT) phenotypes were evaluated.

**Results:**

ERBB2 mRNA amplification was found in HCC datasets, and its expression was downregulated in high grade HCC with a worse overall survival. HER2 overexpression was identified in H4IIE, HepG2, JM1 cells, and 82% (14/17) HCC samples, and tumor stage was correlated with expression of HER2, E‐cadherin, and Vimentin (*P* < 0.05). Trastuzumab with the high concentrations suppressed proliferation of HER2‐positive hepatoma cells (*P* < 0.05); in the coculture model to induce EMT of JM1 cells, HER2 expression increased with downregulated E‐cadherin and upregulated Vimentin. Trastuzumab intravenous injection inhibited in vivo tumor size and metastases (*P* < 0.05). Signal analysis revealed that HER2 functioned through upregulation of β‐catenin and inhibition of SMAD3.

**Conclusion:**

HER2 expression pattern is linked with tumor stage and overall survival; the transforming function of HER2 is found more relevant through β‐catenin and SMAD3. HER2‐targeted treatment is recommended to suppress the HER2‐mediated tumor growth during postoperative liver regeneration.

## 1 INTRODUCTION

Hepatocellular carcinoma (HCC) ranks among the most prevalent cancers and leading causes of cancer deaths worldwide, accounting for about 750 000 deaths per year.^1^, ^2^ Liver surgery provides the only curative treatment of HCC with early stage. Despite successful surgery achieving R0 resection and an achieved long‐term survival, tumor recurrence after surgery especially in patients with advanced HCC is a major problem and might be 50%‐70% at 5‐year observation.^3^, ^4^ Most HCC patients are diagnosed at advanced stages, and generally HCC at this stage is associated with poor prognosis.^5^ SHARP trial^6^ and RESORCE trial^7^ showed that Sorafenib and Regorafenib afford an overall survival gain of these HCC patients or postoperative patients with tumor recurrence by delaying HCC progression up to 3 months. Despite that the advance of genomic prediction identifies more etiologic markers and therapeutic targets of HCC, more efforts are desperately needed to clarify the exact function and molecular mechanisms of HCC biology, providing the novel target for precise cancer therapy.

Human Epidermal Growth Factor Receptor 2 (HER2) is a member of the epidermal growth factor receptor which is involved in the transmission of proliferation and differentiation signals.^8^, ^9^ HER2 overexpression has been documented in some cancer types, and is associated with worse biologic behavior and poor prognosis.^9^, ^10^ The literature contains conflicting data regarding HER2 expression, and the clinical significance of HER2 in HCC is still ambiguous. In the past three decades, the most studies on HER2 expression in HCC were prone to claim that HER2 is rarely overexpressed in HCC and may not play an essential role in the process of HCC.^11^, ^12^, ^13^, ^14^, ^15^ Alternatively, other studies have demonstrated that HER2 expression was found elevated in 30%‐40% of HCCs and may be an independent prognostic factor.^16^, ^17^, ^18^, ^19^ HER2 expression and its function in HCC development needs further explorations.

Despite that HER2 might be upregulated in the transformed state of hepatocyte, HER2 is found not expressed in the normal liver and adult primary hepatocytes, or not appreciably induced in the regenerating liver after partial hepatectomy.^20^ Enlightening the expression of HER2 and its further mechanism in HCC could be a new target as a treatment option for HCC as well as tumor recurrence after surgery.

## 2 MATERIALS AND METHODS

### 2.1 Gene expression data and clinical data of HCC patients from the dataset

The ERBB2 (HER2 encoding gene) expression patterns in normal liver tissues and HCC tissues were compared from TCGA HCC cohort, GSE89377 and GSE115018. The clinico‐pathological characteristics of TCGA HCC cohort were summarized in Table S1 and two gene expression datasets, GSE89377 and GSE115018 were described in Supplemental Materials and Methods.

### 2.2 Clinical data and study population

Seventeen patients with HCC receiving hepatectomy or liver transplantation at our department from May 2015 to October 2016 were included in this study. The inclusion criteria, the exclusion criteria, clinical characteristics, and pathologic findings of the 17 HCC patients were described in Supplemental Materials and Methods and Table S2.

### 2.3 Determination of HER2 expression in hepatoma cells and HCC‐related samples

In order to determine the status of HER2 expression in HCC, we selected hepatoma cell lines (H4IIE, HepG2, JM1, McA‐RH7777) and the archive materials of 17 HCC patients with immunohistochemistry (IHC) and western blotting (WB). Breast cancer sample, which was confirmed with positive HER2 expression from the pathology report, and H4IIE hepatoma cell line, which was reported HER2 positive,^20^ were used as positive control. And rat normal liver tissue, which was reported HER2 negative,^20^ was regarded as negative control*.*

To induce epithelial‐to‐mesenchymal transition (EMT) of hepatoma cell, JM1 cells (JM1/C) were coincubated with transforming growth factor beta (TGF‐β, 0.5 ng/mL, MyBioScience, San Diego, CA) or vehicle for indicated number (n: JM1/n) of passages (JM1/6+).^21^ Hepatoma cells grouped with the naïve cells (JM1/C) and the cocultured cells (JM1/1+ and JM1/6+) were used for further measurements. Tumor cells were harvested for further investigation of the expression of HER2, E‐cadherin, Vimentin, β‐catenin, CD133, and MMP‐9.

### 2.4 Tumor cell inoculation and animal surgical model

Inbred male Fischer 344 rats (NHsd; Harlan Laboratories, Boxmeer, Netherlands), weighing 230‐280 g and aged 9‐12 weeks, were used for the experiments. In vivo inoculation of JM1/C hepatoma cells in the synergetic Fischer 344 rat was modified from the 70% hepatectomy model.^21^ After inoculation of tumor cells and surgery, animals were randomly grouped into the treatment group (Group T, N = 5) receiving Trastuzumab with 8 mg/kg as intravenous infusion through tail vein and the control group (Group C, N = 5) receiving saline. When animals were euthanized 21 days after hepatectomy, tumor volume and the presence of metastasis were recorded and compared in between the treatment group and the control group.

### 2.5 Immunostaining analysis (IHC and WB) and evaluation of IHC

IHC and WB were performed, and immunohistochemical staining was determined as previously described.^22^ Antibodies against HER2, pERBB2, E‐cadherin, Vimentin, AKT, pAKT, ERK, pERK, β‐catenin, SMAD2/3, pSMAD2/3, pSMAD2, CD133, MMP‐9, β‐tubulin, and anti‐β‐catenin activity were described in Supplemental Materials and Methods *.*

### 2.6 Cell proliferation assay (MTT assay)

Hepatoma cells (4  10^5^ cells per well) were grown in 6‐well plates overnight and then treated with various concentrations of Trastuzumab (low concentrations of 0 and 10 g/mL, and high concentrations of 30 and 100 g/mL) for 48 hours. Trypan blue staining confirmed >90% cell viability, and cell numbers were determined by MTT.^23^

### 2.7 Transient transfection

Transit transfection of McA cells was performed with LipofectAMINE 2000 according to the manufacturer's protocol. Transfection was described and characterized in Supplemental Materials and Methods *.*

### 2.8 Statistics

Kaplan‐Meier analysis was used to analysis the association of tumorous ERBB2‐expression with the prognosis of HCC patients. Differences between two groups were analyzed by the two‐tailed Student's *t* test and Wilcoxon signed‐rank test, and differences of more than two groups by one‐way analysis of variance (ANOVA). The association between the tumor expressions of HER2, E‐cadherin, Vimentin, and tumor stage was analyzed with the nonparametric correlation Spearman correlation analysis. The statistical tests were employed by using SPSS version 16.0 (IBM, Armonk, New York, NY), and the results for *t *test, ANOVA, and Spearman correlation were denoted as *t, F, and *correlation coefficient. A probability level of less than 5% (*P* < 0.05) was considered statistically significant.

## 3 RESULTS

### 3.1 ERBB2 mRNA declines with increasing HCC grade and predicts worse overall survival

**Figure 1:**
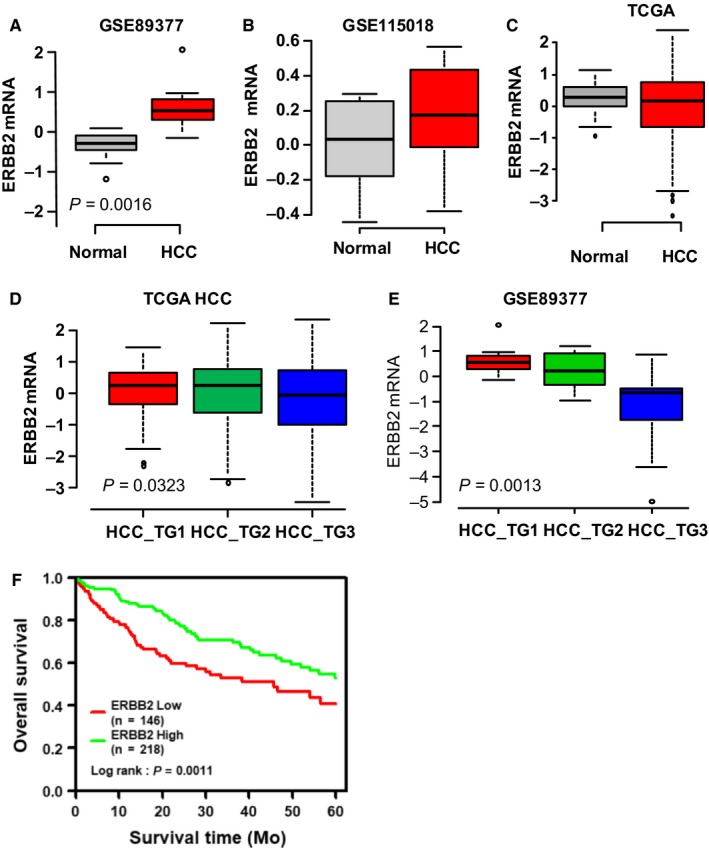
Correlation of ERBB2 mRNA expression and hepatocellular carcinoma (HCC) progression. A‐C. The expression of ERBB2 mRNA in normal and malignant liver tissues was compared and analyzed in the gene expression datasets (TCGA HCC cohort, GSE115018 and GSE89377). D‐E. The correlation of ERBB2 mRNA expression and HCC grade was analyzed in TCGA dataset and GSE89377 dataset. F. The association of ERBB2 mRNA with the survival of HCC patients was evaluated by Kaplan‐Meier analysis in the TCGA cohort.

We firstly analyzed the expression of ERBB2 mRNA in normal and malignant liver tissues in three public gene expression datasets of TCGA HCC cohort, GSE89377 and GSE115018. The results showed an increased median level of ERBB2 mRNA expression in HCC tissues compared with normal liver tissues in GSE89377 (*P* = 0.0016, Figure 1A) and GSE115018 (*P* = 0.128, Figure 1B), but not in the TCGA cohort (Figure 1C).

Next we analyzed the correlation between ERBB2 and various clinico‐pathological characteristics, including gender, age (<55 and ≥55 years), TNM stage, risk factors (HBV infection, HCV infection, nonalcoholic fatty liver disease), and alcohol abuse, using the TCGA cohort (Table S1). No significant correlation was found between ERBB2 mRNA expression and most of the characteristics but tumor grade (Figure S1). The result showed that ERBB2 mRNA expression declined significantly along with increasing tumor grade in the TCGA cohort (*P* = 0.0323) and GSE89377 dataset (*P* = 0.0013, Figure 1D‐E). In accordance with this correlation, Kaplan‐Meier analysis showed a low tumorous ERBB2‐expression was significantly associated with a poor prognosis with within a 5‐year observation (N = 364, *P* = 0.0011, Figure 1F).

### 3.2 HER2 protein is overexpressed in both HCC cell lines and HCC tissues, and HER2 expression pattern correlates with tumor stage and phenotypes of EMT

**Figure 2:**
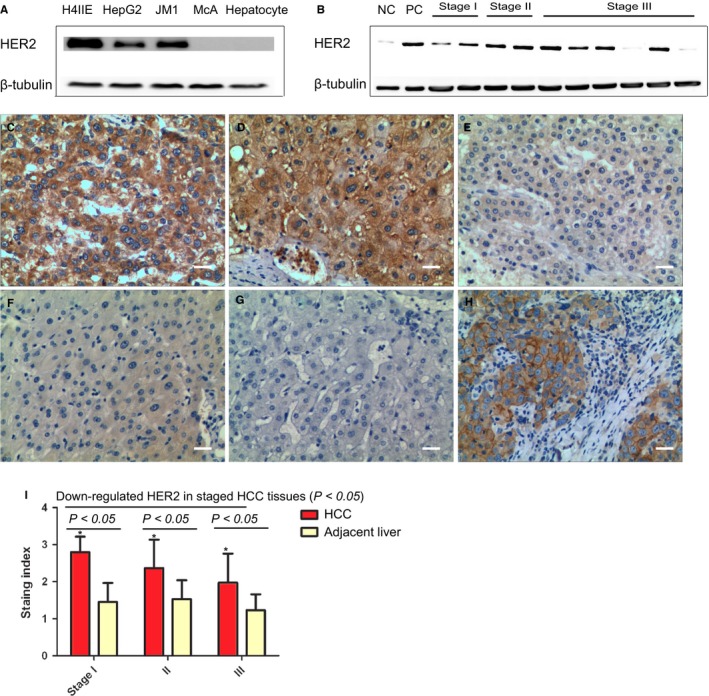
Overexpression of HER2 in hepatocellular carcinoma (HCC) tissue. A. Expression of HER2 in human HCC cell lines in human (H4IIE and HepG2), rat HCC cell lines (JM1 and McA) and rat primary hepatocytes with WB. H4IIE cell and rat primary hepatocytes were regarded as positive control and negative control respectively. B. Expression of HER2 in surgically resected HCC tissue samples by WB (*N* = 12). Normal liver tissue and resected HER2‐positive breast cancer tissue were regarded as negative control (NC) and positive control (PC) respectively. C‐H. Representative photographs of strongly positive staining (+++, C and D) of HER2 protein and weakly positive staining (+, E) with IHC in HCC tissue (magnification × 200 and scale bars 20 μm). Representative photographs of negative staining and weakly positive staining (−~−/+) of HER2 protein in the adjacent peritumoral liver tissue (F) and normal liver tissue (G). Resected HER2‐positive breast cancer tissue was regarded as positive control (++, H). I. HER2 expression increased in HCC tumor tissue compared with the adjacent liver tissue, and down‐regulated expression of HER2 in the increased stages of liver cancer tissue with a semi‐quantitative immunostaining score (*P < 0.05).

As discrepancy was observed when comparing ERBB2 mRNA expression between normal and tumor tissues (Figure 1A‐C), we next assessed the HER2 protein in both hepatoma cells and the resected HCC tissues with WB and IHC. The results showed that HER2 was widely overexpressed in both hepatoma cells and the resected HCC samples, while HER2 was low‐expressed in primary hepatocytes^20^ and the adjacent normal liver from the resected HCC patients (Figure 2A‐B). Interestingly, in contrast to membranous HER2 overexpression in the breast cancer as positive control, a significant proportion of HCC (82%, 14/17) showed both cytoplasmic and membranous overexpression (Figure 2C‐H).

The semi‐quantification of IHC indicated a significant upregulation of HER2 of HCC patients compared with the adjacent liver (*t* = 9.218, 4.973 and 4.919, *P* = 0.001, Figure 2I). Besides, consistent with the observation of HER2 expression pattern from GSE89377, HER2 expression in stage I (100%, 4/4), II (83%, 5/6), and III (71%, 5/7) showed a downregulation along with the progression of the tumor stage (*F* = 8.879, *P* = 0.001). Spearman correlation analysis indicated that tumor stage was negatively correlated with HER2 expression (correlation coefficient −0.423, *P* = 0.001).

**Figure 3:**
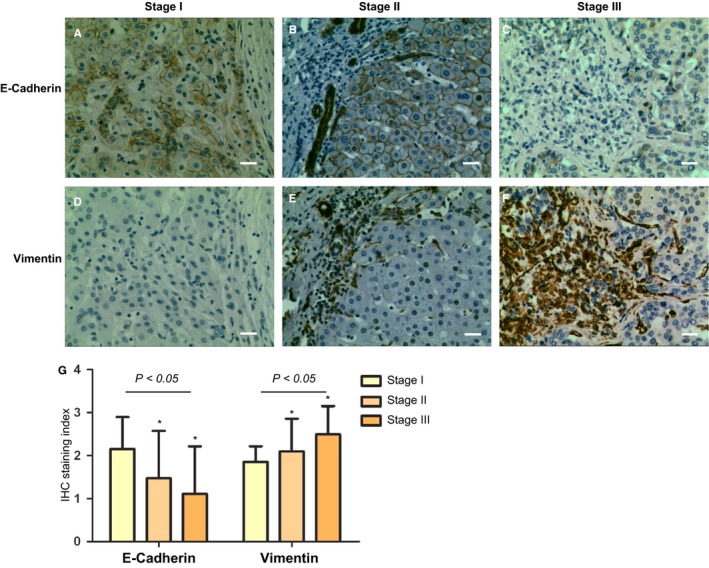
Expression of epithelial‐to‐mesenchymal transition phenotypes (E‐cadherin and Vimentin) in hepatocellular carcinoma (HCC) tissue. A‐F. Representative photographs of positive staining of E‐cadherin protein (A, B, C) and positive staining of Vimentin protein (D, E, F) in a *serial sections* of ` × 200 and scale bars 20 μm). G. The relationship of *E‐cadherin, vimentin* and tumor stage was confirmed with a semi‐quantitative immunostaining score (*versus data of stage I, *P* < 0.05).

Tumorous expression of E‐cadherin and Vimentin was found a significant difference from stage I to stage III (N = 17, *F* = 6.869 and 6.763, *P* = 0.002) with semi‐quantification of IHC (Figure 3A‐G). In addition, there was a negative correlation of E‐cadherin expression with tumor stage (correlation coefficient −0.364, *P* = 0.001), while there was a positive correlation between Vimentin staining and tumor stage (correlation coefficient 0.398, *P* = 0.001). Correlation analysis of the tumor stage and expressions of HER2, E‐cadherin, and Vimentin indicated that regulation of HER2 expression is related with EMT phenotypes of and tumor progression.

### 3.3 HER2 is related with in vitro and in vivo proliferation and EMT of HCC

To further explore the function of HER2, both HER2‐transfection in HER2‐negative expressed McA cells and monoclonal antibody targeting HER2, Trastuzumab, were applied in HCC cells, including HepG2, JM1, and HER2‐transfected McA cells, to test the effect of HER2 on biological characteristics.

**Figure 4:**
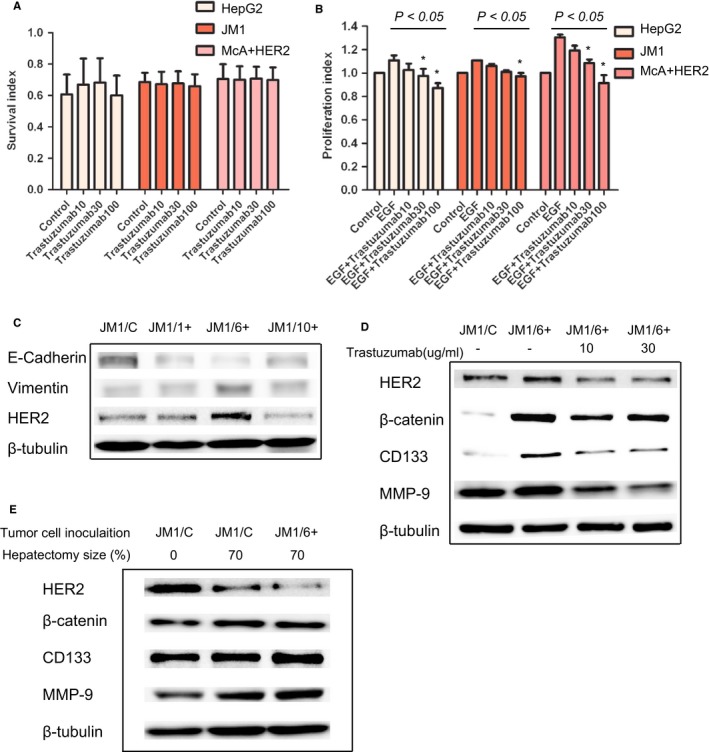
Expression of HER2 and survival, proliferation, phenotypes of epithelial‐to‐mesenchymal transition (EMT), stemness and invasiveness of HER2‐positive hepatoma cells. A. Effect of HER2 inhibition on the survival of HepG2, JM1 and HER2‐transfected McA cells. B. HER2 inhibition can decrease proliferation of HepG2, JM1 and HER2‐transfected McA cells. Trastuzumab with the concentrations of 30 and 100 μg/ml suppressed cell proliferation after 48 hours of EGF stimulation (*versus data of control, *P* < 0.05). C. The decreased level of E‐cadherin and the increased level of Vimentin indicates EMT in JM1 with TGF‐β (0.5μg/ml) for 40 days. HER2 expression follows pattern of Vimentin or mesenchymal character of transit. D. Expression of HER2, β‐catenin, CD133 and MMP‐9 in both the transformed JM1 cells (JM/6+) and naïve JM1 cells, and effect of HER2 inhibition on deceased level of β‐catenin, CD133 and MMP‐9.

Firstly, Trastuzumab within the concentration of between 10 and 100 g/mL was employed for 48 hours to test the cell survival with MTT assay. Results showed that Trastuzumab treatment did not confer to a survival decrease in HCC tumor cells (*F* = 0.082361, 0.022506, and 0.002358, *P* = 0.968, 0.995, and 0.999) (Figure 4A).

Since the specific ligand to HER2 receptor is unidentified,^24^ and HER2 may function as didiemer more with EGFR in HepG2 cells,^25^ cell proliferation ability was assessed through EGF stimulation in serum free medium. Trastuzumab with the high concentrations of 30 and 100 g/mL suppressed slightly (10%‐20%) the proliferation of HepG2, JM1, and HER2‐transfected McA cells, (*F* = 3.422, 17.174, and 10.001, *P* = 0.029, 0.001, and 0.001) (Figure 4B), indicating HER2 may function as a proliferation receptor through receptor dimerization with EGFR after EGF stimulation.

In the coculture model to induce EMT in JM1 cells, WB analysis found upregulated HER2 expression along with downregulated E‐cadherin and upregulated Vimentin, especially in the JM1/6+ cells (Figure 4C), indicating that regulation of HER2 may be associated with tumor EMT. Furthermore, based on the previous study,^21^ expression of β‐catenin, CD133, and MMP‐9 in JM1/6+ cells was upregulated when compared with JM1/C cells. Trastuzumab with the low concentrations of 10 and 30 g/mL suppressed the expression of HER2, β‐catenin, CD133, and MMP‐9 in JM1/6+ cells, which justified the contribution of HER2 to EMT (Figure 4D).

Previous studies have demonstrated that HER2 overexpression leads to an increased invasion in in vitro matrigel assays.^26^ Our previous study^21^ indicated an increased growth and invasion of the in vivo tumor through EMT during the liver regeneration. In order to further determine whether HER2 may affect tumor growth and invasion, Trastuzumab was injected in Fischer 344 rats with JM1 inoculation. Twenty‐one days after tumor inoculation and hepatectomy, there was a significant decrease in the tumor size and metastases of the animals in Group T, compared with those animals in Group C (*t* = 6.132 and 3.464, *P* = 0.001 and 0.009, Table S3). The results indicated that Trastuzumab, through downregulation of HER2 at early stage, might inhibit tumor growth and metastasis in the in vivo microenvironment.

### 3.4 HER2 mediates cell signaling through β‐catenin and SMAD3 pathways

**Figure 5:**
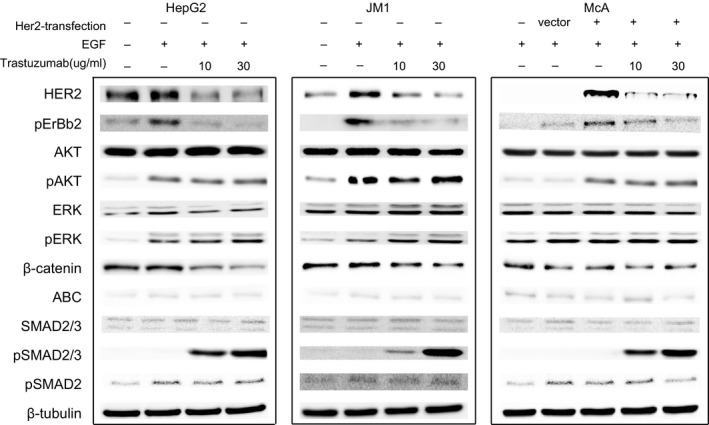
HER2‐mediated signaling pathways in HER2‐positive hepatoma cells. Effect of HER2 inhibitor, Transtuzumab, on the activation and expression of the major signal molecular at 10 min after 10 nM EGF stimulation in HepG2, JM1 and McA+HER2 assessed by Western blotting. β‐tubulin was set as the internal control. The result indicated that HER2 may affect the biological characteristics of hepatoma cells through both TGF‐β/SMAD2/3 and WNT/β‐catenin signal pathways. HER2 degradation through Transtuzumab may function as β‐catenin suppression and SMAD3 and SMAD2/3 complex activation.

Since HER2 may function as heterodiemerization with EGFR, HER2‐mediated cellular signaling pathways were assessed through EGF stimulation. After transfection of HER2 in McA cells, phosphorylated levels of AKT and ERK were increased at 10 minutes after EGF stimulation. In HER2‐overexpressed hepatoma cells, HepG2, JM1, and HER2‐transfected McA cells, phosphorylated levels of HER2, AKT, ERK, and β‐catenin were increased at 10 minutes after EGF stimulation (Figure 5). Application of 10 and 30 g/mL Trastuzumab inhibited phosphorylated HER2 and β‐catenin, and phosphorylated levels of AKT, ERK, and SMAD2 were unchanged; phosphorylated level of SMAD2/3 was increased in a dose‐dependent manner (Figure 5). After pretreatment with TGF‐β type I receptor inhibitor SD‐208,^27^ the upregulation of phosphorylated level of SMAD2/3 was not suppressed.

The findings above indicated that HER2 may function through upregulation of β‐catenin and inhibition of SMAD2/3 complex after EGF stimulation, while activation of AKT and ERK is independent of HER2. HER2 degradation by Trastuzumab may function through β‐catenin suppression and activation of SMAD3 independent of TGF‐β receptor.

## 4 DISCUSSION

Immunostaining was used for HER2 protein detection expression in HCC tissues in most of the articles where HER2 protein was claimed not detectable^15^. With IHC and WB, our results showed that HER2 protein is overexpressed in hepatoma cell lines of H4IIE, HepG2, JM1, and 82% (14/17) of HCC resected tissues. This result was supported by a recent bioinformatics analysis on differentially expressed genes between liver cancer samples and normal samples, which identified ERBB2 as one of top 10 key genes related with HCC initiation and progression^28^ In addition, the status that HER2 expression in the tumor correlates negatively with the tumor stage was supported with both the gene analysis of GSE89377 and TCGA HCC cohort, and the immunostaining analysis of the patient samples and clinical data through the correlation analysis of HER2 expression pattern and the tumor stage.

Discrepancy in the pattern of ERBB2 mRNA expression between normal and malignant liver tissues was observed in the HCC cohorts from independent studies (Figure 1). The reason of this discrepancy is not clear. Based on our immunostaining analysis of HER2 expression and the tumor stage, the different outcomes from three datasets may be related with the patient selection of different HCC stages. Most of HCC patients are diagnosed with advanced stage at the first diagnosis^1^, ^2^ The more patients with higher tumor stages and the lower HER2 expression in general, might account for no significant change of ERBB2 mRNA between liver cancer and normal liver in TCGA cohort. The same reason may also account for the greater value of median level of ERBB2 mRNA in HCC tissues than normal liver tissues in GSE115018, while the difference did not reach the degree of significance based on the data analysis from the 12 HCC patients. In addition, although our analysis from the TCGA HCC cohort indicated that ERBB2 mRNA expression in the tumor has no correlation with HBV or HCV infection, alcohol abuse, liver cirrhosis, NAFLD, data on HER2‐overexpression in HCC patients need verification from more cases with different etiology of liver diseases.

As to the function of HER2 in HCC tumorgenesis, our analysis suggested that regulation of HER2 may affect the epithelial‐to‐mesenchymal transforming more than tumor cell survival, and the later has been highlighted in the breast cancer and other tumor types.^9^, ^10^ Intravenous Trastuzumab infusion decreased in vivo tumor growth significantly, which may partly be related with Trastuzumab, a humanized monoclonal antibody, which may invoke systematic unspecific response and affect the inoculated tumor growth.

The analysis of phenotype transit and signaling molecular indicated that HER2 may function through upregulation of β‐catenin and inhibition of SMAD2/3 complex activation. The functional connection among HER2, β‐catenin, and SMAD3 was supported with the evidence that HER2 and β‐catenin are physically associated.^29^, ^30^ Immunoprecipitation of HER2 in normal and transformed epithelial cells was coimmunoprecipitated with the cadherin‐catenin complex, revealing a HER2 and β‐catenin in association with the basal condition*.* 29, 30 The connection between HER2 and SMAD3 independent of TGF‐β receptor might be regulated through β‐catenin/Smad3 colocalization.^31^, ^32^

Based on these findings, function of HER2 in HCC tumorgenesis and progression is defined as: (1) Amplification of ERBB2 gene (Figure 1A,B) confers an early event of HCC tumorgenesis, and HER2 overexpression declines with increasing HCC grade (Figures 1E and 2); (2) In HER2‐positive HCC subtype, HER2 may affect tumor growth and invasion through increased heterodimerization with other EGF receptor members, and β‐catenin/SMAD3 pathways; (3) Tumor progression might be mediated through regulation of HER2 and EMT (Figures 2 and 3), and a low tumorous ERBB2‐expression was associated with a higher tumor stage and a poor prognosis (Figure 1F). HER2 inhibition at early stage might inhibit tumor progression through suppression of growth and metastasis. Regarding that HER2 is found not expressed in the normal liver nor induced in the regenerating liver after partial hepatectomy,^20^ our animal experiment evidenced that application of HER2 inhibition after partial hepatectomy could inhibit tumor growth thus avoiding tumor recurrence.

The ERBB2 oncogene hypothesis, that overexpression of HER2 correlates etiologically with tumorgenesis,^33^ has been challenged by some studies claiming that HER2 was rarely overexpressed in HCC or might not play an essential role in the process of HCC.^11^, ^12^, ^13^, ^14^, ^15^ Our study supported the hypothesis with the evidences of HER2 expression and its function in HCC. In addition, the therapeutic application of HER2 inhibition in the preclinical model of partial hepatectomy could impede HCC progression in the postoperative period. Thus HER2‐targeted treatment has potential utility to suppress the HER2‐mediated tumor growth, especially during postoperative liver regeneration.

## CONFLICTS OF INTEREST

The authors declare that there is no conflict of interest regarding the publication of this paper.
