# Corrigendum

**DOI:** 10.1002/cam4.3567

**Published:** 2020-11-28

## Abstract

Recognition of HER2 expression in hepatocellular carcinoma and its significance in postoperative tumor recurrence.

To the Editor:

We notice an unintentional error in our published article “Recognition of HER2 expression in hepatocellular carcinoma and its significance in postoperative tumor recurrence”.^1^ The Figure 5 comprised the images of HER2‐mediated signaling pathways in HER2‐positive hepatoma cells. Some images of pAKT and pSMAD2 of HepG2 and McA were selected by mistake.

The major finding of this figure was to show both pSMAD2/3 and β‐catenin might affect the biological characteristics of HER2‐positive hepatoma cells by western blotting. Since AKT, ERK, and SMAD2 alone did not affect the biological characteristics of hepatoma cells but were regarded as the negative effectors, this error does not change the scientific conclusions of the article. We have attached a corrected version of Figure 5 after deleting the negative results.

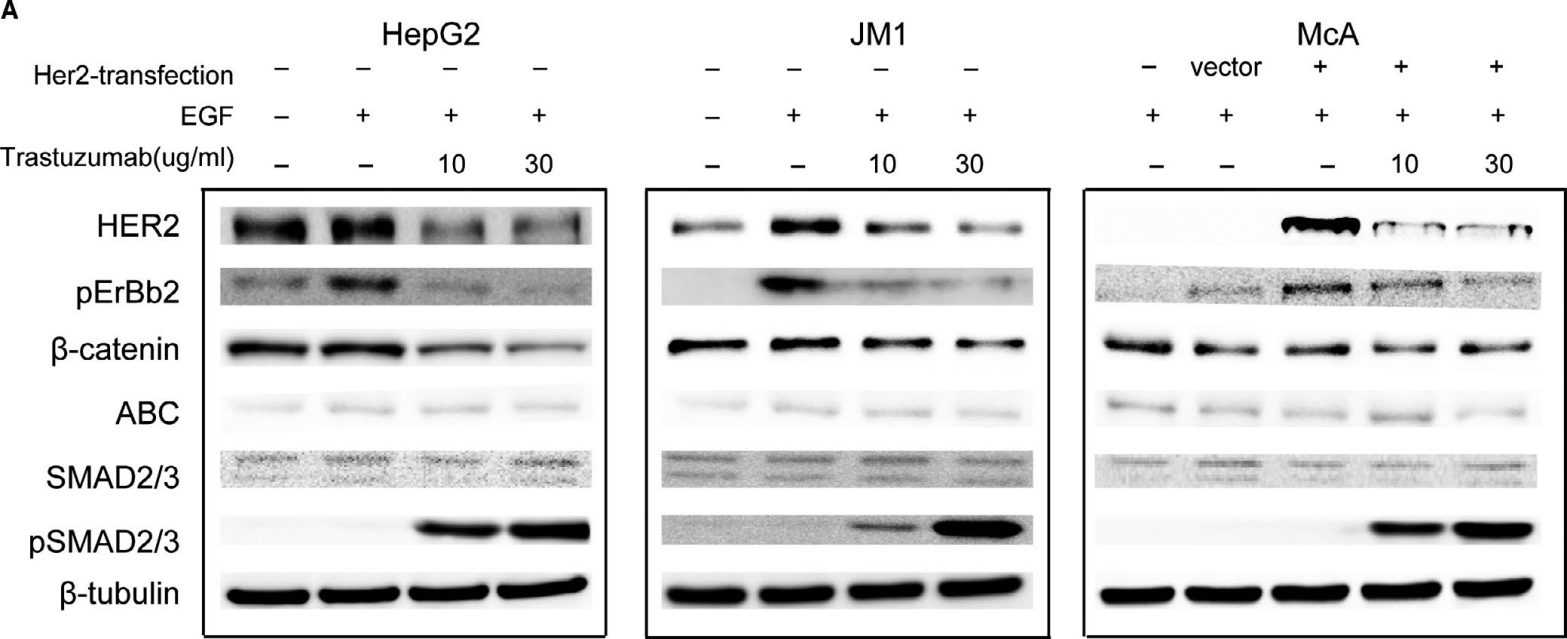

The authors sincerely apologize for this error.

Ji‐Hua Shi, Wen‐Zhi Guo, Yang Jin, Huapeng Zhang, Chun Pang, Jie Li, Pål‐Dag Line, Shui‐Jun Zhang
