# Supplementary figures and images for: Recognition of HER2 expression in hepatocellular carcinoma and its significance in postoperative tumor recurrence
Source: Cancer Med. 2019 Feb 4;8(3):1269–78. doi: 10.1002/cam4.2006 (PMC6434216; doi:10.1002/cam4.2006)

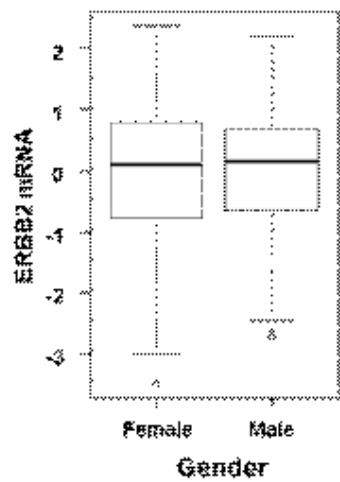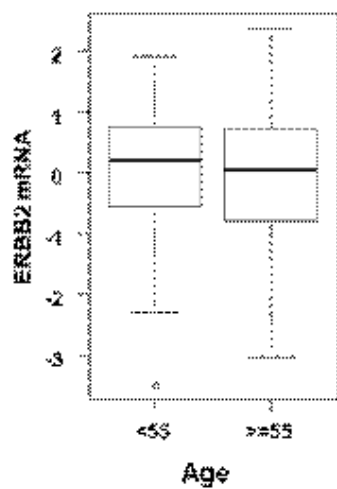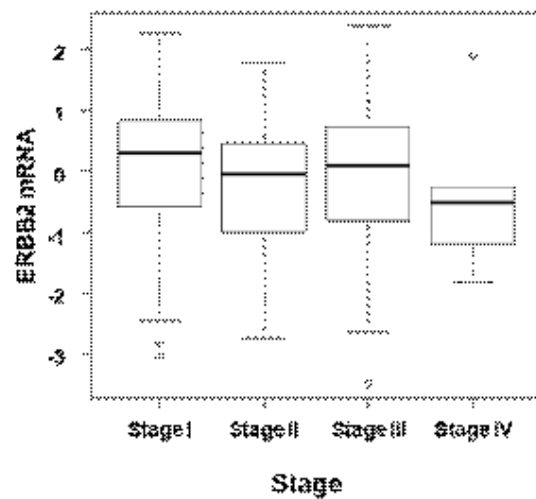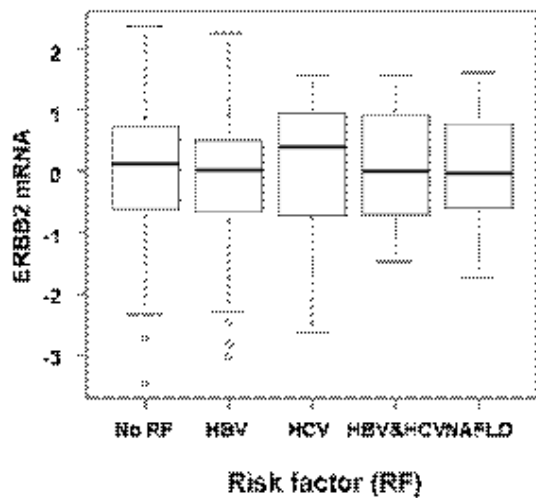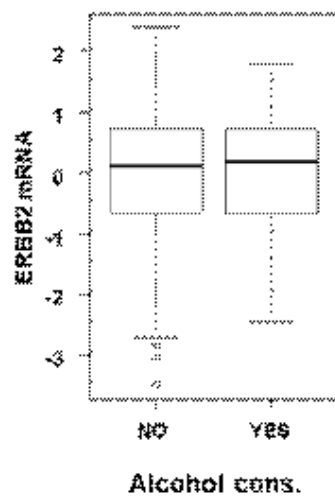

Supplement: Supplementary file 1 [file CAM4-8-1269-s001.pdf]
