# Supplementary material for: Recognition of HER2 expression in hepatocellular carcinoma and its significance in postoperative tumor recurrence
Source: Cancer Med. 2019 Feb 4;8(3):1269–78. doi: 10.1002/cam4.2006 (PMC6434216; doi:10.1002/cam4.2006)
Supplement: Supplementary file 2 [file CAM4-8-1269-s002.pdf]

Evolution of HCC

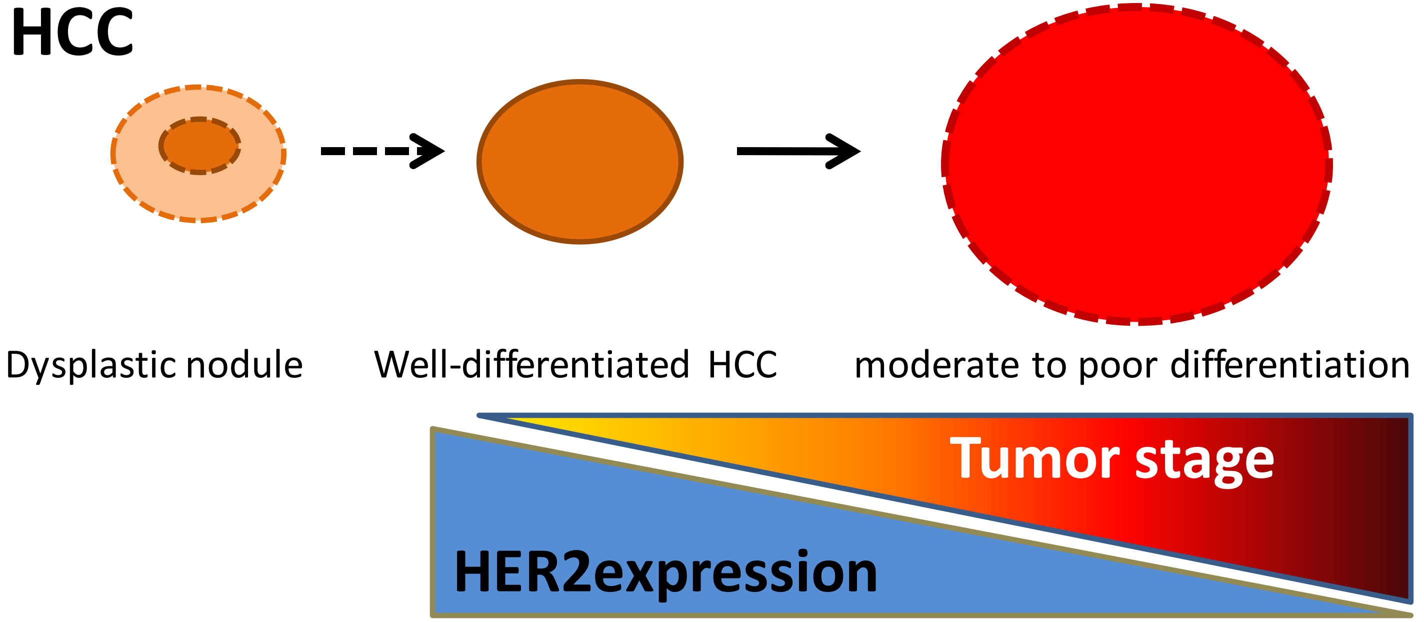

HER2 inbitition treatment

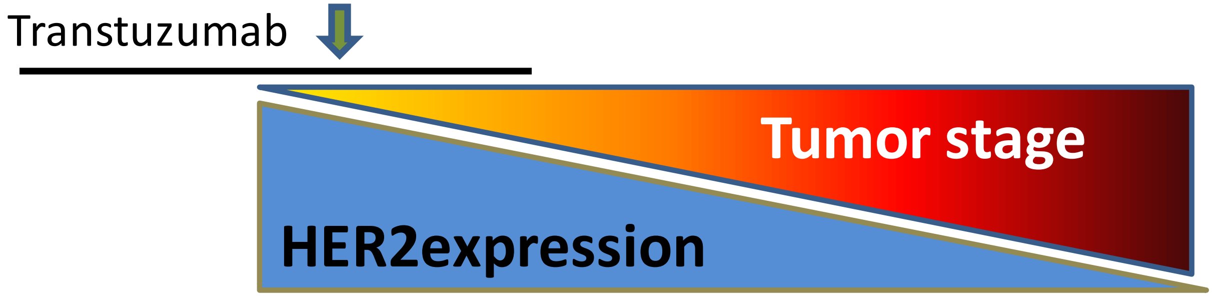

1. *In vitro* HER2 inhibition
with Transtuzumab in HER2-positive HCC cells (Figure 3,4)

Cell survival	Cell proliferation	EMT
-	↓ (10-20%)	↓↓↓

2. *In vivo* HER2 inhibitition
with Transtuzumab in intrahepatic tumor in rats (Table S3)

Tumor size	Metastasis
↓↓ (40%)	↓↓↓(60%)
