# Supplementary material for: Recognition of HER2 expression in hepatocellular carcinoma and its significance in postoperative tumor recurrence
Source: Cancer Med. 2019 Feb 4;8(3):1269–78. doi: 10.1002/cam4.2006 (PMC6434216; doi:10.1002/cam4.2006)
Supplement: Supplementary file 3 [file CAM4-8-1269-s003.docx]

| **Table S1.** Patient characteristics (as provided by TCGA) | |
| --- | --- |
|  |  |
| Total number of HCC patients | 371 |
| Male gender (%) | 240 (67.6 %) |
| Number of matched non-HCC patients | 50 |
|  |  |
| Age (>55) | 129 (34.7%) |
|  |  |
| Ethnicity (%) |  |
| American Indian or Alaska native | 1 (0.3 %) |
| Asian | 156 (44.8 %) |
| Black or African American | 17 (4.9 %) |
| White | 171 (49.1 %) |
| Not evaluated | 3 (0.9 %) |
|  |  |
| Etiology (%) |  |
| Unknown | 78 (24.9 %) |
| Alcohol | 77 (24.6 %) |
| Alcohol and Hepatitis | 15 (4.8 %) |
| Hepatitis B | 100 (31.9 %) |
| Hepatitis C | 33 (10.5 %) |
| NASH | 10 (3.2 %) |
|  |  |
| Stage (%) |  |
| I | 164 (49.2 %) |
| II | 82 (24.6 %) |
| III and IV | 87 (26.1 %) |
|  |  |
| Residual tumor (%) |  |
| R0 | 313 (89.7 %) |
| R1 | 15 (4.3 %) |
| R2 | 1 (0.3 %) |
| RX | 20 (5.7 %) |
|  |  |
| Pathologic nodes (%) |  |
| N0 | 247 (69.8 %) |
| N1 | 4 (1.1 %) |
| NX | 103 (29.1 %) |
|  |  |
| Metastasis (%) |  |
| M0 | 260 (73.2 %) |
| M1 | 4 (1.1 %) |
| MX | 91 (25.6 v) |
|  |  |
| Vascular invasion (%) |  |
| None | 196 (65.3 %) |
| Micro | 90 (30.0 %) |
| Macro | 14 (4.7 %) |

**Table S2**. Clinico-pathologic features of 17 HCC patients

|  | **Age** (yrs) | **Gender** | **HBV infection** | **Liver cirrhosis** | **Serum AFP level** (μg/L) | **TNM/AJCC Stage** | **Child-pugh stage** | |
| --- | --- | --- | --- | --- | --- | --- | --- | --- |
| **No. 1** | 48 | Male | Positive | Positive | <400 | 1 | B |  |
| **No. 2** | 59 | Male | Positive | Positive | <400 | 1 | A |  |
| **No. 3** | 34 | Male | Positive | Positive | <400 | 1 | A |  |
| **No. 4** | 51 | Male | Positive | Positive | <400 | 1 | B |  |
| **No. 5** | 57 | Male | Positive | Positive | <400 | 2 | A |  |
| **No. 6** | 35 | Male | Positive | Positive | <400 | 2 | A |  |
| **No. 7** | 55 | Male | Positive | Positive | <400 | 2 | A |  |
| **No. 8** | 51 | Male | Positive | Positive | >400 | 2 | A |  |
| **No. 9** | 43 | Male | Positive | Positive | <400 | 2 | A |  |
| **No. 10** | 51 | Male | Positive | Positive | <400 | 2 | A |  |
| **No. 11** | 42 | Male | Positive | Positive | <400 | 3 | A |  |
| **No. 12** | 53 | Male | Positive | Positive | <400 | 3 | A |  |
| **No. 13** | 56 | Male | Positive | Positive | <400 | 3 | A |  |
| **No. 14** | 54 | Male | Positive | Positive | <400 | 3 | A |  |
| **No. 15** | 50 | Female | Positive | Positive | >400 | 3 | A |  |
| **No. 16** | 50 | Male | Positive | Positive | <400 | 3 | A |  |
| **No. 17** | 46 | Male | Positive | Positive | <400 | 3 | A |  |

**Table S3.** Tumor development *in vivo* after Trastuzumab injection.

|  | | **Group C** (n=5) | **Group T** (n=5) |
| --- | --- | --- | --- |
| **Tumor volume** (mm^3^) | | 1320.0±532.2 | 769.6±423.8* |
| **Metastasis**  (n/N) |  | 5/5 | 2/5* |
|  | local metastasis | 2/5 | 2/5 |
|  | distant metastasis | 3/5 | 0/5 |

* *P*<0.05 compared with Group C.
