# Supplementary material for: Recognition of HER2 expression in hepatocellular carcinoma and its significance in postoperative tumor recurrence
Source: Cancer Med. 2019 Feb 4;8(3):1269–78. doi: 10.1002/cam4.2006 (PMC6434216; doi:10.1002/cam4.2006)
Supplement: Supplementary file 4 [file CAM4-8-1269-s004.docx]

**Supplemental materials and methods**

**Gene expression data and clinical data of HCC patients from the dataset**

### HCC cohort was downloaded from cBioportal ([www.cbioportal.org/](http://www.cbioportal.org/)). The gene expression dataset includes mRNA-Seq data of 371 HCC samples and 50 normal liver samples. The clinico-pathological characteristics including age, gender, histological grade, TNM stage, overall survival information and the known risk factors (hepatitis virus status and alcohol abuse) were summarized in Table S1. The ERBB2 (HER2 encoding gene) expression patterns in normal liver tissues and HCC tissues were compared in two additional gene expression datasets, GSE89377 and GSE115018. The GSE89377 dataset includes 13 normal liver samples and HCC samples of 3 tumor grades (9 of TG1, 12 of TG2 and 14 of TG3) (Eun et al., unpublished). The GSE115018 dataset includes 12 normal liver samples and 12 HCC samples without information of tumor grade (Ye et al., unpublished).

### Clinical data and study population

This study was approved by the Ethics Review Committee of the First Affiliated Hospital of Zhengzhou University. A number of seventeen patients with HCC receiving hepatectomy or liver transplantation at our department from May 2015 to October 2016 were included in this study (Table S2). The inclusion criteria were patients: 1) with tumor-node-metastasis (TNM) stage I, II, III according to the eighth edition of the tumor node metastasis (TNM)/American Joint Committee on Cancer (AJCC) staging system[^1^](#_ENREF_1); 2) with Child-Pugh class A and class B hepatic function; 3) aged 18–80; and 4) provided written informed consent. The exclusion criteria included patients: 1) with TNM stage IIIC, IV; 2) with Child-Pugh class C hepatic function; 3) with second malignancy or history of second malignancy within 5 years; 4) with perioperative dysfunction of vital organs; 5) with percutaneous ablation, 6) transcatheter arterial chemoembolization; and 7) received chemotherapy, or radiotherapy within 1 month post-operation. All patients were postoperatively followed-up as routine. Normal liver tissues, paired HCC and tumor-adjacent tissues were collected were collected and prepared as described in previous study[^2^](#_ENREF_2). Clinical data, including gender, age, HBV infection, liver cirrhosis, serum AFP level, tumor size, tumor number and TNM/AJCC stage were collected from the patients’ records in hospital. Clinical characteristics and pathologic findings of the 17 HCC patients were described in Table S2.

**Hepatoma cell culture**

The hepatoma cell lines included HepG2 and McA-RH7777 (McA) that were obtained from American Type Culture Collection, and JM1 that was kindly donated by Dr George Michalopoulos at University of Pittsburgh. HepG2, JM1 and McA were maintained in Dulbecco’s modified Eagle’s medium supplemented with 10% (v/v) fetal bovine serum, 2 mM L-glutamine, and 1% penicillin/streptomycin.

**Tumor cell inoculation and animal model**

All the animal experiments were approved by Norwegian Animal Research Authority (FOTS project number 2806), and were performed in accordance with both Norwegian Animal Welfare Act and European legislation concerning animal experimentation. *In vivo* inoculation of JM1/C hepatoma cells in the synergetic Fischer344 rat was modified from the previous hepatectomy model ^[3](#_ENREF_3)^. Intrahepatic injection of 0.5х10^6^ tumor cells (JM1/C) in 0.1 ml PBS was performed in the right lobe, and 70% hepatectomy was performed with removal of both left and median lobes of rats. When animals were euthanized on 21 days after hepatectomy, tumor volume and the presence of metastasis were recorded and compared in between the treatment group and the control group. Tumor volume was calculated using the formula: Volume = L х W^2^/2 (L: length of the tumor and W: width of the tumor) as before[^3^](#_ENREF_3).

### Immunohistochemical analysis and evaluation of Immunohistochemical staining

IHC was performed as previously described [^4^](#_ENREF_4). Monoclonal anti-ERBB2 antibody (1:150 dilution, Thermo Fisher Scientific, Rockford, USA), anti-cadherin antibody (1:100 dilution, Cell Signaling Technology, Beverly, USA ) and anti-Vimentin antibody (1:100 dilution, Cell Signaling Technology, Beverly, USA) was used in the immunohistochemical analysis.

The immunohistochemical staining in the tissue was determined independently by 2 pathologists blinded to the patients and clinical data using Nikon ECLIPSE Ni*-*E400 microscope (Nikon, Tokyo, Japan) and NIS Elements Basic Research Microscope Imaging Software, and staining index was quantified in 5 random visual fields (200×) with a semiquantitative immunoreactivity score [^2^](#_ENREF_2). Category A documented the intensity of immunostaining as 0–3 (0, negative; 1, weak; 2, moderate; 3, strong). Category B documented the percentage of immunoreactive cells as 0 (less than 5%), 1 (6%–25%), 2 (26%–50%), 3 (51%–75%), and 4 (76%–100%). Multiplication of category A and B resulted in an immunoreactivity score ranging from 0 to 12 for each tumor or non-tumor. Sections with a total score of 0 or 1 or 2 were defined as negative (−), score of 3 or 4 were defined as weakly positive (+), score of 6 or 8 were defined as moderately positive (++), score of 9 or 12 were defined as strongly positive (+++). For categorical analyses, the immunoreactivity was graded as low level (total score ≤4) or high level (total score > 4).

**WB**

Anti-ERBB2 antibody (1:1000 dilution, Thermo Fisher Scientific, Rockford, USA) and 1:1000 dilution, ABCam, Cambridge, UK), pERBB2 (1:1000, phospho Y877,ABCam, Cambridge, UK), AKT (1:1000 dilution, Cell Signaling Technology, Beverly, USA), pAKT (1:1000 dilution, Cell Signaling Technology, Beverly, USA), ERK (1:1,000, Santa Cruz Biotechnology, Santa Cruz, USA), pERK (1:1000 dilution, Cell Signaling Technology, Beverly, USA), β-catenin (1:1000, ABCam, Cambridge, UK), anti-β-catenin activity (1:1000 dilution, ABC, clone 8E7, Millipore-Upstate, Charlottesville, USA), SMAD2/3 (1:1000 dilution, Cell Signaling Technology, Beverly, USA), pSMAD2/3 (1:1000 dilution, Cell Signaling Technology, Beverly, USA), pSMAD2 (1:1000 dilution, Cell Signaling Technology, Beverly, USA), β-tubulin (1:5000, Sigma Aldrich, St. Louis, USA), CD133 (1:1000, ABCam, Cambridge, UK), MMP-9 (1:1000, ABCam, Cambridge, UK), HRP-conjugated secondary antibodies against Mouse IgG (1:5000, Jackson ImmunoResearch Laboratories Inc., West Grove, USA) and Rabbit IgG (1:5000, Jackson ImmunoResearch Laboratories Inc., West Grove, USA) was used in the immunohistochemical analysis (1:200 dilution).The immunoreactive signals were visualized by ECL plus Western blotting detection reagents (Little Chalfont, Amersham, UK) and detected by scanning densitometry with ChemiDoc™ Touch Imaging System (Bio-Rad Laboratories, Inc. Hercules, USA).

**Transient transfection**

Rat HER2/neu gene with GFP tag was a generous gift from Prof. Henrik S. Huitfeldt of Instittute of Pathology at Oslo University Hospital (Oslo, Norway). Transit transfection of McA cells with either cDNA-HER2 or vector was performed with LipofectAMINE 2000 (Invitrogen, Life Technologies, Rockford, USA) according to the manufacturer's protocol. The transfected McA cells were characterized for expression of HER-2/neu gene with WB and immunofluorescence of GFP tag mediated by the monoclonal anti-HER-2 antibody (1:1000 dilution, Thermo Fisher Scientific, Rockford, USA and 1:1000 dilution, ABCam, Cambridge, UK).

References

**1.** Amin MB, Greene FL, Edge SB, et al. The Eighth Edition AJCC Cancer Staging Manual: Continuing to build a bridge from a population-based to a more “personalized” approach to cancer staging. *CA: a cancer journal for clinicians.* 2017;67(2):93-99.

**2.** Li GQ, Guo WZ, Zhang Y, et al. Suppression of BRD4 inhibits human hepatocellular carcinoma by repressing MYC and enhancing BIM expression. *Oncotarget.* Jan 19 2016;7(3):2462-2474.

**3.** Shi JH, Scholz H, Huitfeldt HS, Line PD. The effect of hepatic progenitor cells on experimental hepatocellular carcinoma in the regenerating liver. *Scand J Gastroenterol.* Jan 2014;49(1):99-108.

**4.** Shi JH, Hammarstrom C, Grzyb K, Line PD. Experimental evaluation of liver regeneration patterns and liver function following ALPPS. *BJS Open.* Jun 2017;1(3):84-96.
